# Association of apolipoprotein M and sphingosine-1-phosphate with brown adipose tissue after cold exposure in humans

**DOI:** 10.1038/s41598-022-21938-2

**Published:** 2022-11-05

**Authors:** Anna Borup, Ida Donkin, Mariëtte R. Boon, Martin Frydland, Borja Martinez-Tellez, Annika Loft, Sune H. Keller, Andreas Kjaer, Jesper Kjaergaard, Christian Hassager, Romain Barrès, Patrick C. N. Rensen, Christina Christoffersen

**Affiliations:** 1 Department of Clinical Biochemistry, Section 3-01-3, Rigshospitalet, Blegdamsvej 9, 2100 Copenhagen, Denmark; 2 Novo Nordisk Foundation Center for Basic Metabolic Research, Faculty of Health and Medical Sciences, University of Copenhagen, Copenhagen, Denmark; 3 Department of Medicine, Division of Endocrinology, and Einthoven Laboratory for Experimental Vascular Medicine, Leiden University Medical Center, Leiden, The Netherlands; 4 Department of Cardiology, Rigshospitalet, Copenhagen, Denmark; 5 Department of Clinical Physiology, Nuclear Medicine & PET and Cluster for Molecular Imaging, Rigshospitalet and University of Copenhagen, Copenhagen, Denmark; 6 Department of Biomedical Sciences, University of Copenhagen, Copenhagen, Denmark; 7 Department of Clinical Biochemistry, Bispebjerg Hospitalet, Copenhagen, Denmark

**Keywords:** Biomarkers, Endocrinology

## Abstract

The HDL-associated apolipoprotein M (apoM) and its ligand sphingosine-1-phosphate (S1P) may control energy metabolism. ApoM deficiency in mice is associated with increased vascular permeability, brown adipose tissue (BAT) mass and activity, and protection against obesity. In the current study, we explored the connection between plasma apoM/S1P levels and parameters of BAT as measured via ^18^F-FDG PET/CT after cold exposure in humans. Fixed (n = 15) vs personalized (n = 20) short-term cooling protocols decreased and increased apoM (− 8.4%, *P* = 0.032 *vs* 15.7%, *P* < 0.0005) and S1P (− 41.0%, *P* < 0.0005 *vs* 19.1%, *P* < 0.005) plasma levels, respectively. Long-term cooling (n = 44) did not affect plasma apoM or S1P levels. Plasma apoM and S1P did not correlate significantly to BAT volume and activity in the individual studies. However, short-term studies combined, showed that increased changes in plasma apoM correlated with BAT metabolic activity (β: 0.44, 95% CI [0.06–0.81], *P* = 0.024) after adjusting for study design but not BAT volume (β: 0.39, 95% CI [− 0.01–0.78], *P* = 0.054). In conclusion, plasma apoM and S1P levels are altered in response to cold exposure and may be linked to changes in BAT metabolic activity but not BAT volume in humans. This contrasts partly with observations in animals and highlights the need for further studies to understand the biological role of apoM/S1P complex in human adipose tissue and lipid metabolism.

## Introduction

Obesity is an increasing worldwide problem^1^ and is due to an imbalance in calorie in- and output causing storage of excess energy as triglycerides in white adipose tissue^2^. Obesity is associated with deleterious consequences such as type 2 diabetes and cardiovascular disease^2^. Exploring regulatory mechanisms of energy metabolism is of high importance to identify new treatment strategies to combat obesity and associated disorders.

Cold exposure leads to increased energy expenditure, peripheral vasoconstriction, and decreased peripheral blood flow to maintain core body temperature^3,4^. Lipids are estimated to fuel 50% of the heat production (*e.g.* thermogenesis) during shivering^5^, and exclusively during non-shivering thermogenesis induced by mild cooling^6^. Mild cold exposure stimulates the sympathetic nervous system and induces a release of norepinephrine, which interacts with beta-adrenergic receptors and activates brown adipose tissue (BAT)^7^. The presence and the metabolic activity of BAT in human adults^8–12^ may be visualized by the uptake of ^18^F-fluorodeoxyglucose (^18^F-FDG) during a positron emission tomography/computed tomography scan (PET/CT)^7^. Under non-shivering cold conditions in humans, BAT takes up glucose and (triglyceride-derived) non-esterified fatty acids (NEFA), which replenishes intracellular triglyceride stores^13,14^. Thus, activation of BAT by cold exposure, by increasing energy expenditure and the utilization of lipid stores, has potential as a treatment that targets excess adiposity and also insulin resistance, as 10 days of cold exposure improves insulin sensitivity in individuals with type 2 diabetes^15^.

The HDL-associated apolipoprotein M (apoM) may affect energy metabolism via regulation of BAT volume, BAT activity, and triglyceride turnover, as demonstrated in mice^16^. ApoM-deficient mice (apoM-KO) are protected against diet-induced obesity and have improved glucose tolerance^16^. ApoM transports approximately 70% of sphingosine-1-phosphate (S1P) in plasma, whereas albumin carries the remaining S1P^17^. Interestingly, treating wild-type mice with S1P-receptor 1-antagonist results in hyperactive BAT similar to apoM-KO mice accompanied by accelerated triglyceride turnover^16^. S1P exerts its effect through five G-protein coupled receptors (S1PR1-5)^18^ and plays also an important role in maintaining the endothelial barrier and vasodilation.

BAT is a highly vascularized tissue due to its high metabolic rate. During cold exposure, the perfusion through BAT is increased to facilitate glucose uptake and BAT perfusion correlates positively with whole-body energy expenditure in preclinical models^19^. The increased perfusion in BAT may lead to increased delivery of other nutrients including (triglyceride-derived) fatty acids to BAT and increased export of generated heat to peripheral tissues. For example, the apoM-KO mice, which show a decrease in S1P levels^17^, displays increased permeability and uptake of triglycerides in BAT^16^. Yet, whether apoM and S1P play a role in BAT activity in humans is unknown. Thus, was the current study aimed to investigate whether short-term cold exposure applied to a minor or larger skin surface area affects plasma S1P, its carrier apoM and whether plasma S1P and apoM are associated with ^18^F-FDG uptake by BAT in cold-exposed humans.

## Results

### Cold exposure in animals

Cold exposure of wild-type mice is associated with increased BAT activity^20^. We have also observed that apoM-deficiency (accompanied by a reduced level of S1P), as well as treatment with S1P-receptor 1 antagonist in wild-type mice, are associated with increased BAT activity and volume^16^. Whether cold per see affects the apoM/S1P metabolism is unknown. To explore whether cold exposure of mice affects plasma S1P and apoM levels, wild-type mice were housed at 23 ℃ or 4 ℃ for 16 h (Supplementary Fig. S1A and B). Plasma S1P and apoM were both increased significantly after cold exposure.

### Basic characteristics of human participants exposed to cold

**Figure 1:**
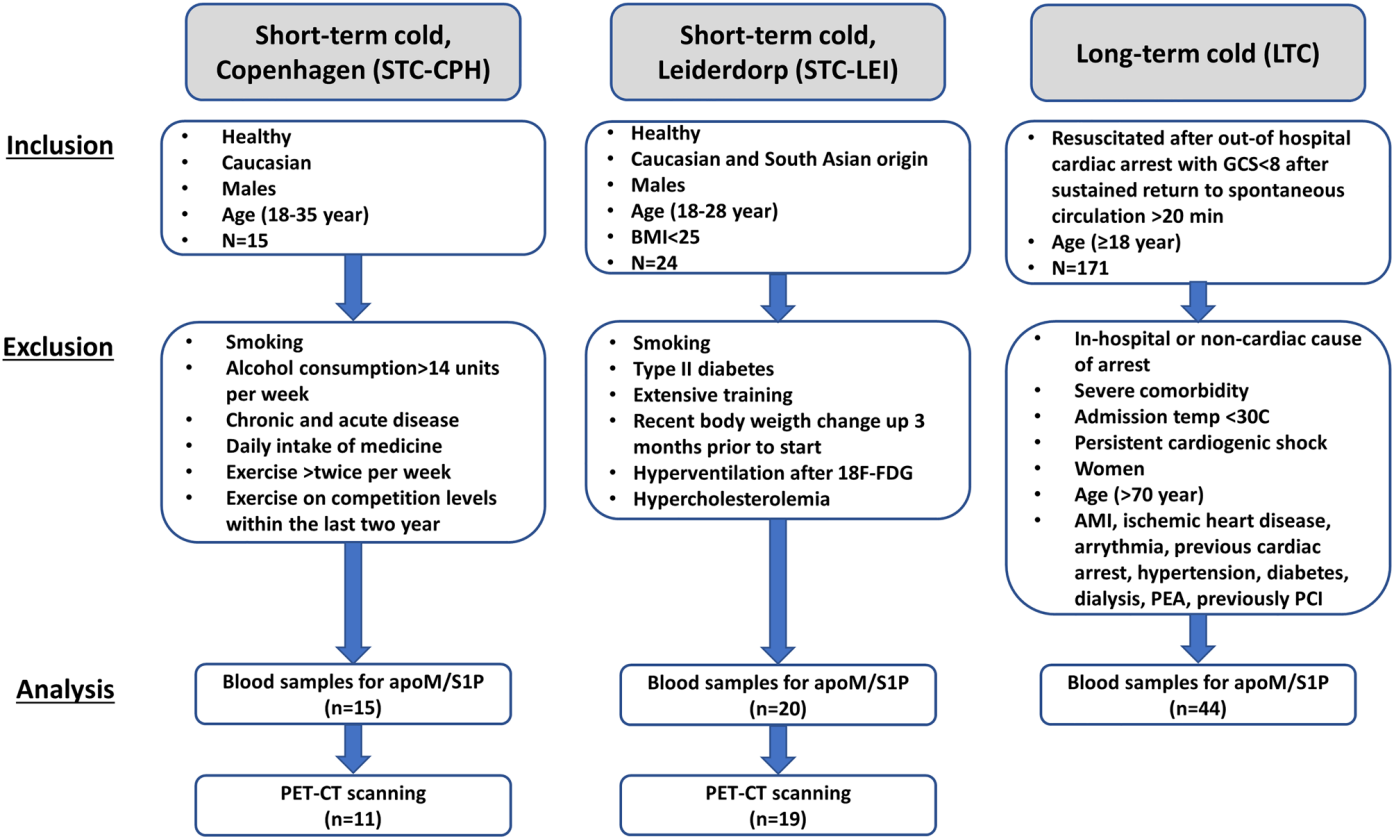
Flow charge of the three included studies and the exclusion criteria.

**Figure 2:**
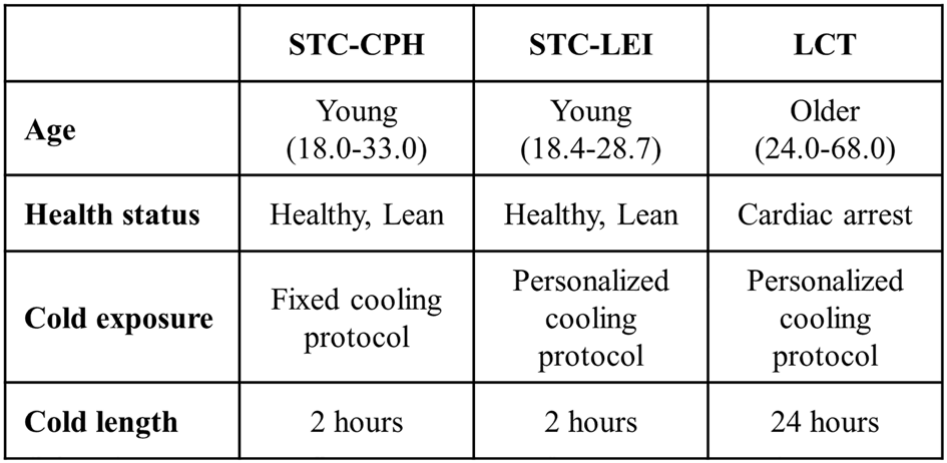
Overview of the three included studies and the difference in age, healt status, cold protocol, and exposure time. STC-CPH; short-term cold, Copenhagen. STC-LEI; short-term cold, Leiderdorp. LTC; long-term cold.

To explore whether apoM/S1P levels were affected by short-term or long-term cold exposure and associated with BAT volume and activity in humans we next included three cohorts (Fig. 1). Two cohorts represent short-term (2 h) cold applied to a minor or larger surface of the body, and one cohort represents long-term (24 h) cold exposure. Basic characteristics are presented in Supplementary Table S1 and an overview of the cohorts is presented in Fig. 2. Short-term cold-Copenhagen (STC-CPH), included 15 healthy young (24.3 ± 4.6 years) white Caucasian males with an average BMI of 23.4 kg/m^2^. The participants were exposed to 2 h fixed cooling protocol on the upper part of the body. Short-term cold-Leiderdorp (STC-LEI) included 20 healthy young males with Dutch white Caucasian and Dutch South Asian ethnicity with an average age of 24.4 ± 2.8 years and BMI of 21.9 kg/m^2^. Cold exposure was applied for 2 h by a personalized cooling protocol applied on both upper and lower parts of the body. The long-term cold (LTC) study included 44 male patients either subjected to a core body temperature of 33 or 36 ℃ for 24 h after cardiac arrest with an average age of 55.7 and 53.5 years, respectively. All patients included in LTC had an initial core body temperature of 35.2 ℃ and 35.3 ℃ for the 33 ℃ and 36 ℃ group, respectively^21^. Hence, the 36 ℃ group was maintained cold, whereas the 33 ℃ group was subjected to intensified cooling to reach a target temperature of 33 ℃.

### Lipid parameters and response to cold

Lipid levels are presented in Supplementary Table S2. In STC-CPH, cold exposure did not affect the lipid parameters total cholesterol (TC), LDL-cholesterol (LDL-C), HDL-cholesterol (HDL-C), or triglycerides (Trig). In STC-LEI, TC increased by 7.8% (*P* = 0.001), LDL-C increased by 7.4% (*P* = 0.006), and Trig by 19.3% (*P* = 0.004), but no differences were observed for HDL-C after cold exposure.

The LTC study included patients exposed to either 33 ℃ or 36 ℃ for 24 h after cardiac arrest. There was no effect of target temperature, only an overall effect of cooling on all lipid parameters. Thus, TC, HDL-C, and LDL-C decreased after 24 h, whereas Trig increased. Further, analyzing the change in the difference between the two temperatures (Δ-values), showed no differences for either lipid parameter (data not shown).

Free fatty acids (FFA) increases in response to cold exposure due to liberation from white adipose tissue (WAT) during lipolysis. In both STC-CPH and STC-LEI, FFA levels were increased, but did only reach statistical significance in STC-LEI (34.1%, *P* < 0.005) (Supplementary Table S3).

### The effect of cold on apoM plasma levels

To explore the relationship between cold and the apoM/S1P complex in humans, we measured plasma apoM and S1P in participants of the three cohorts.

**Figure 3:**
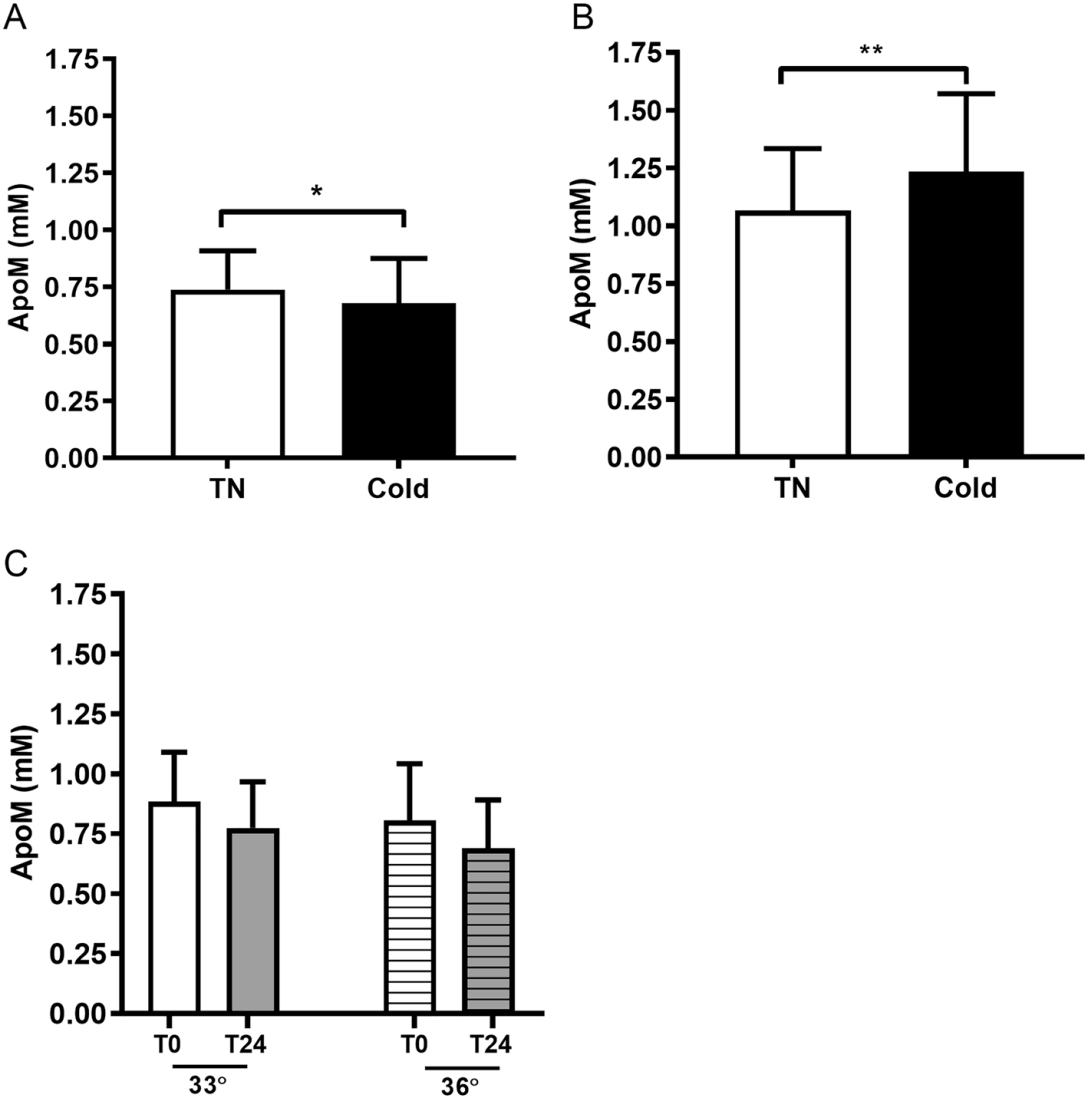
ApoM plasma levels in STC-CPH (A), STC-LEI (B), and LTC (C). Thermoneutral (TN) and cold refers to pre-cooling and after cold exposure (2 h) in STC-CPH (n = 15) and STC-LEI (n = 20). In LTC samples were taken at 0 and after 24 h at either 33˚C (n = 20, intensified cold) or 36˚C (n = 24, cold maintenance). Data are presented as mean with error bars as SD. In A and B, data are analyzed with a paired t-test or Wilcoxon signed-rank test depending on normal distribution. In C, data are analyzed with a two-way mixed ANOVA with time and temperature as variables. *P < 0.05, **P < 0.01, ***P < 0.0005 for TN vs Cold. STC-CPH; short-term cold, Copenhagen. STC-LEI; short-term cold, Leiderdorp. LTC; long-term cold.

Short-term cold exposure in STC-CPH decreased plasma apoM by 8.4% (*P* = 0.032) (Fig. 3A). In contrast, apoM levels were increased by 15.7% (*P* < 0.0005) in participants in STC-LEI (Fig. 3B). The increase in apoM levels was similar between ethnicity (data not shown).

In the LTC study, there was no effect of target temperature on plasma apoM, but only an effect of cooling (*P* < 0.0005, Fig. 3C.). Furthermore, Δ-values (pre-cooling versus cooling) within each temperature group were not significantly changed (data not shown).

ApoM physically interacts with lipoproteins and thus correlates mainly with TC and HDL-C^22,23^. Therefore, differences within these lipid parameters could have caused the opposed changes in plasma apoM in the present studies. Thus, the ratio between either plasma apoM to TC or HDL-C was also reported. In STC-CPH the difference between apoM at thermoneutral (pre-cooling) and cold maintained significant after correcting for HDL-C (*P* = 0.02) but not TC (Supplementary Fig. S2A and B). In STC-LEI, the difference between apoM at thermoneutral and cold maintained significant after correcting for both TC and HDL-C (*P* = 0.031 and *P* = 0.0006, respectively) (Supplementary Fig. S2C and D). In the LTC study, the decrease in apoM levels after cold exposure was not maintained after correction for HDL-C or TC (Supplementary Fig. S2E and F).

### The effect of cold on plasma S1P levels

**Figure 4:**
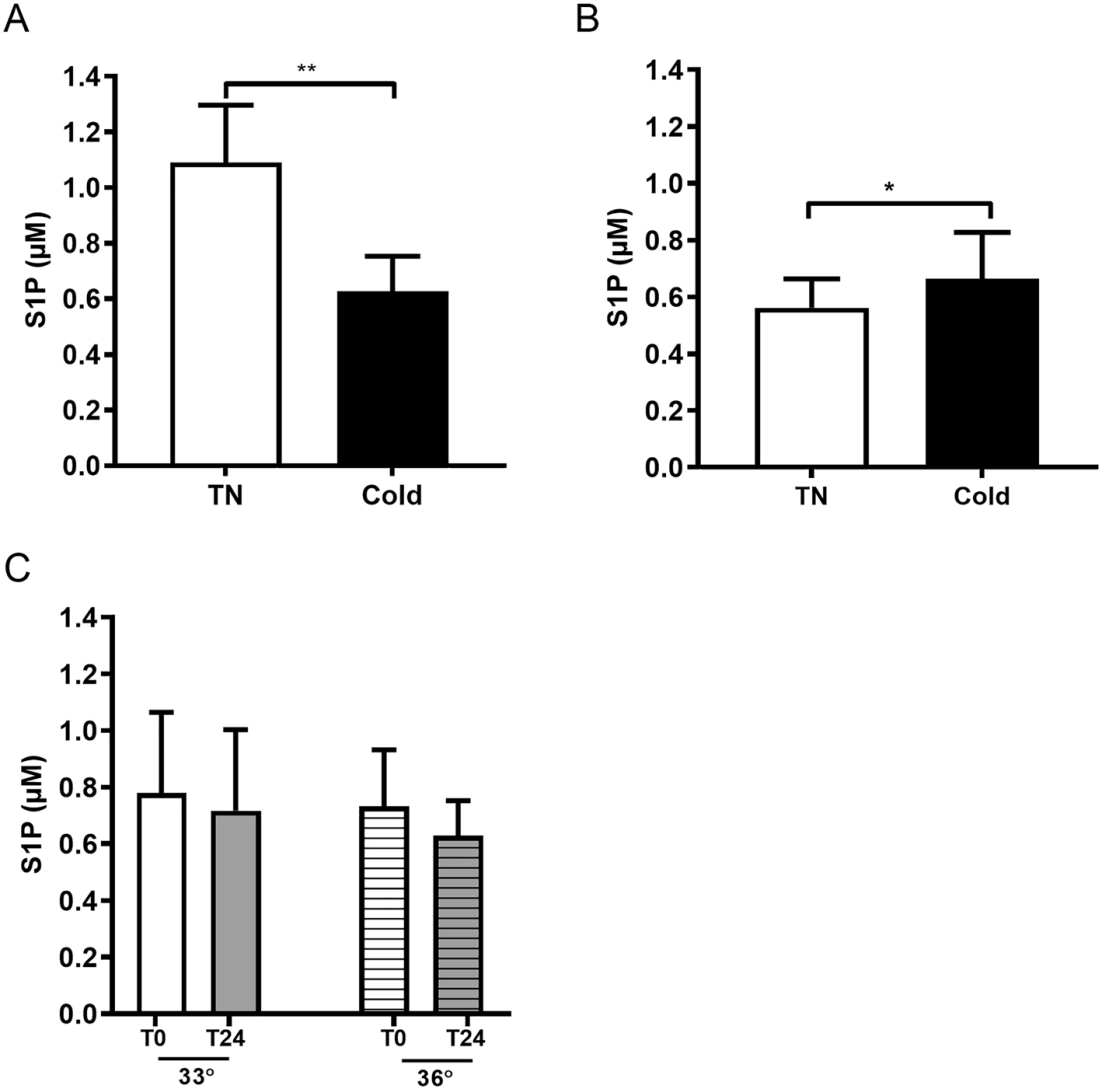
S1P plasma levels in STC-CPH (A), STC-LEI (B), and LTC (C). Thermoneutral (TN) and cold refers to pre-cooling and after cold exposure (2 h) in STC-CPH (n = 15) and STC-LEI (n = 20). In LTC samples were taken at 0 and after 24 h at either 33˚C (n = 20, intensified cold) or 36˚C (n = 24, cold maintenance). Data presented as mean with error bars as SD and in A and B and analyzed with either a paired t-test or Wilcoxon signed-rank test depending on a normal distribution. In C data are analyzed with a two-way mixed ANOVA with time and temperature as variables. *P < 0.05, **P < 0.01, ***P < 0.0005 for TN vs Cold. STC-CPH; short-term cold, Copenhagen. STC-LEI; short-term cold, Leiderdorp. LTC; long-term cold.

Short-term cold exposure in STC-CPH decreased plasma S1P levels by 15.9% (*P* < 0.0005) (Fig. 4A.), whereas an increase of 19.1% (*P* = 0.004) was found in STC-LEI (Fig. 4B.).

In the LTC study, there was no significant interaction between the target temperature and cooling, thus only an effect of cooling was found (*P* = 0.019) (Fig. 4C.). Δ-values for time within each temperature group were not significant (data not shown).

In STC-CPH the difference between S1P at thermoneutral and cold maintained significant after correcting for both TC (*P* < 0.0001) and HDL-C (*P* < 0.0001) (Supplementary Fig. S3A and B). In STC-LEI, the difference between S1P at thermoneutral and cold maintained significant after correcting for HDL-C (*P* = 0.01) but not TC (Supplementary Fig. S3C and D). In the LTC study, no effect of cooling was maintained after correction for TC or HDL-C (Supplementary Fig. S3E and F).

### Plasma apoM and S1P and its association with BAT parameters

**Figure 5:**
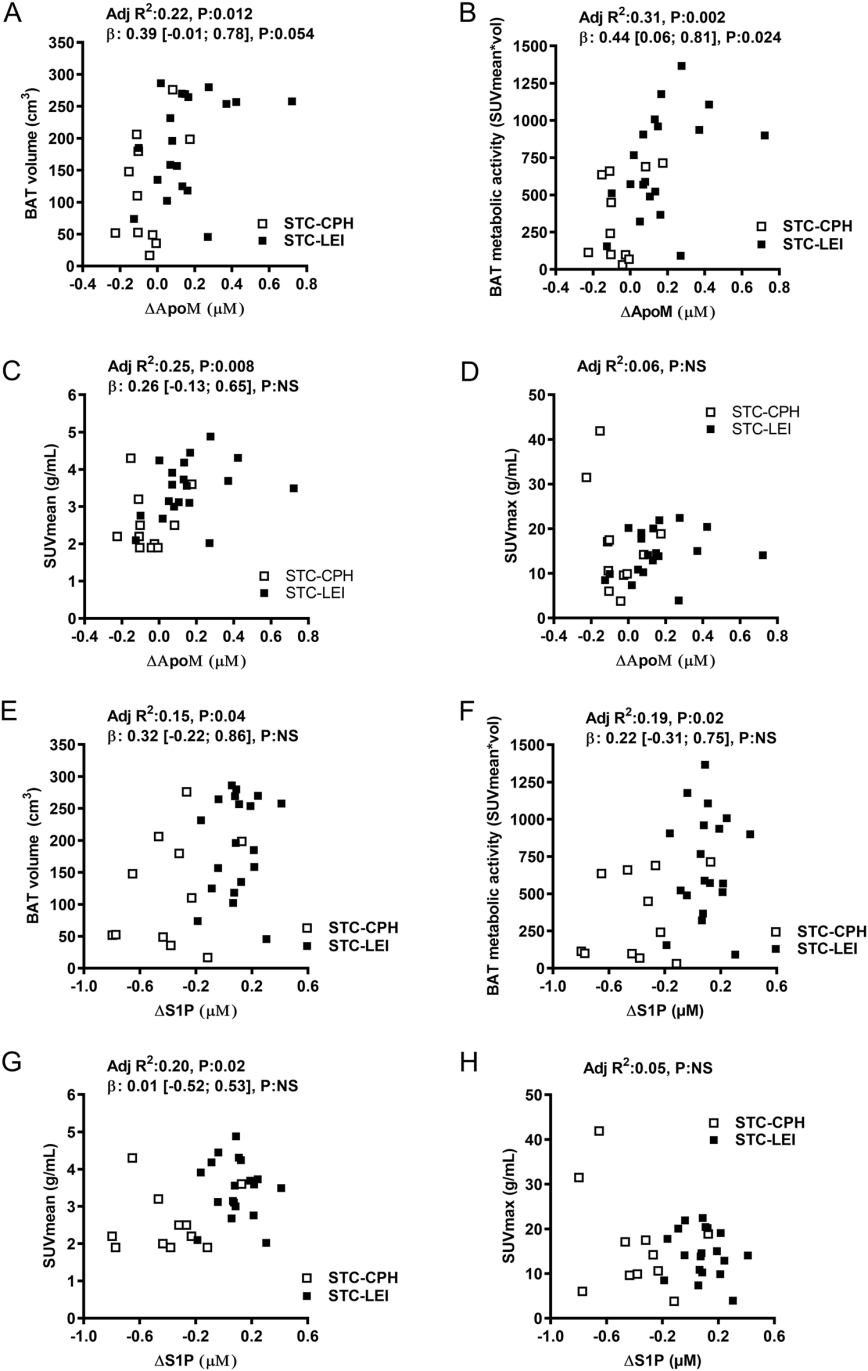
Association between brown adipose tissue (BAT) parameters and delta plasma apoM and S1P levels (ΔapoM and ΔS1P). Delta plasma apoM and S1P levels (cold-induced changes in plasma concentration) were associated with BAT volume (A and E), metabolic activity (B and F), SUVmean (C and G) and SUVmax (D and H). Data were tested for homogeneity before combining the studies in linear regression analysis adjusting for a study place. Adj R^2^; adjusted R-square value. β; beta-coefficient. []; 95%-confident interval. STC-CPH (n = 11); short-term cold, Copenhagen. STC-LEI (n = 19); short-term cold, Leiderdorp.

To explore the association between the apoM/S1P axis and activity and volume of BAT during short-term cold exposure, participants in STC-CPH and STC-LEI were examined. BAT parameters are presented in Table 1. There was no significant association between plasma apoM or S1P with BAT volume, SUVmean or SUVmax in neither the STC-CPH nor STC-LEI (Supplementary Table S4). Combining the two studies after testing for homogeneity however, suggest that cold-induced changes in plasma apoM associate positively with BAT metabolic activity (Fig. 5). The association was maintained after adjusting for study design (β:0.44, 95% CI [0.06–0.81], *P* = 0.024 and adjusted R^2^ of 0.31, *P* = 0.002). No association between plasma apoM or S1P and BAT volume, SUVmean, or SUVmax was observed after adjusting for study design.

**Table 1:** BAT activity and volume after short term cold exposure.

Study	SUV mean (g/mL)	SUV max (g/mL)	BAT activity (SUV mean × vol)	BAT volume (cm^3^)
**STC-CPH**				
All (n = 11)	2.4 (0.7)	15. 5 (11.5)	294.7 (261.2)	109.0 (83.1)
**STC-LEI**				
All (n = 19)	3.6 (0.7)	15.1 (5.2)	729.9 (325.4)	199.5 (71.5)

Data are presented as mean (SD). SUV; g/mL, BAT volume; cm^3^, BAT activity; SUV mean × BAT volume. STC-CPH; short-term cold, Copenhagen. STC-LEI; short-term cold, Leiderdorp.

## Discussion

The present study aimed to investigate the effects of cold exposure on apoM and S1P plasma levels as well as to explore whether the plasma apoM and S1P concentrations correlate with BAT volume and activity in humans. We found that both apoM and S1P plasma levels, as well as lipids, were affected by cold exposure. This effect was depending on the applied cooling protocol, fixed versus personalized. Finally, we observed that BAT metabolic activity correlated positively with cold-induced changes in apoM plasma levels only when both STC studies were combined.

Changes in plasma apoM and S1P levels during short-term cold exposure in healthy lean individuals may reflect changes in production or utilization. ApoM is mainly produced in the liver and kidney in humans^24,25^, while a minor part may be produced by white adipocytes^26^. In addition to activating BAT, cold exposure also results in sympathetic activation of other organs including WAT^27^. Thus, β-adrenergic activation could influence apoM secretion from WAT. Whether such stimulus also triggers the secretion of apoM/S1P from the liver or the kidney is unknown. Clearance of apoM could be another mechanism for regulating plasma apoM/S1P levels. ApoM is mainly associated with HDL particles, but can rapidly exchange onto LDL/VLDL particles^28,29^. Therefore, a high fractional catabolic rate of LDL in humans is related to low plasma apoM concentration^28–30^. Thus, changes in plasma lipids could affect plasma apoM. In the present study, lean healthy men exposed to short-term cold (fixed protocol, STC-CPH) via a cooling vest covering the torso showed no cold-induced differences in lipid levels, but a decrease of 8% and 42% in apoM and S1P plasma levels, respectively. In contrast, similar individuals exposed to whole-body short-term cold (personalized protocol, STC-LEI) showed an increase in lipid parameters and apoM and S1P plasma levels (16% and 18%, respectively). This effect was maintained also after correcting for changes in lipid levels. Thus, the two studies, despite comparable participants regarding BMI, age, and gender, cold exposure affected apoM and S1P levels in opposite direction. An explanation for these differences may be the cooling protocol used in STC-CPH versus STC-LEI. Careful examination of pre-analytical as well as analytical procedures was also considered but no discrepancies were observed between the two studies.

S1P is mainly carried by apoM, but 30–35% is bound to albumin^31,32^. S1P can be produced by various cells such as platelets, erythrocytes, and endothelial cells^33^. Whether any of these cells are affected by cooling and thereby contribute to cold-induced changes in plasma S1P is unknown. Moreover, apoM not only carries S1P but also stimulates the secretion of S1P from erythrocytes. A reduction or an increase in plasma apoM may therefore also directly cause a concomitant change in plasma S1P. Finally, a portion of S1P can bind to albumin, like FFA^34^. Although not significantly increased in STC-CPH, FFA could compete with S1P for binding to albumin resulting in the removal of S1P from the circulation. Alternatively, S1P-albumin could serve as a pool delivering S1P to apoM-containing HDL. The exchange of S1P between carriers and the function of S1P-albumin as an S1P-reservoir is currently unexplored.

The changes in apoM/S1P levels can affect vascular integrity and metabolism as shown in several animal models^16,17^. Hence, apoM-S1P complex bound to HDL maintains the endothelial barrier function^17,35–37^. Furthermore, HDL-S1P induces eNOS production leading to vasodilation^38^, but S1P also suppresses vascular leakage by promoting endothelial barrier integrity^17,39^. ApoM-KO mice have increased permeability in BAT, larger BAT volume, increased metabolism of triglyceride, and improved glucose tolerance compared to WT mice properly conveyed by a decrease in S1P activation of S1Pr1 on endothelial cells^16^. Thus, a decrease in the apoM/S1P levels in humans may lead to increased energy metabolism and the amount of BAT. We found a correlation between greater cold-induced changes in plasma apoM and BAT metabolic activity in healthy, lean human male subjects undergoing 2 h of cold exposure (combined STC-CPH and STC-LEI). Surprisingly, the correlation was positive, thus an increase in plasma apoM after short-term cold exposure was associated with a higher metabolic activity of BAT. Unexpected, but in support of our human data, we also observed an increase of plasma S1P and apoM in wild-type mice after 16 h of cold exposure. These observations do not directly align with the phenotype observed in apoM-KO mice or wild-type mice treated with S1P-receptor 1 antagonist previously reported^16^. In these two animal models lack of apoM, reduced plasma S1P levels, or decreased S1P-receptor 1 activity was associated with increased BAT volume and activity. This may be explained by several mechanisms. The apoM-KO mouse has a life-long deficiency of apoM and a build-up of hyperactive BAT whereas the human participants or wild-type mice in the current study have no known alterations in plasma apoM levels at baseline. In wild-type mice as well as in the human participants we observed a parallel change in apoM and S1P, suggesting that the individuals have a balanced apoM/S1P ratio. This is not the case in apoM-KO mice where S1P mainly is attached to albumin and therefore could affect the S1P-receptors differently compared to apoM-carried S1P. Thus, it is likely that the discrepancy between the present study and the previous relies on changes and shifts in the apoM/S1P versus albumin/S1P ratios. Also, we present correlations in our human studies, and more research is needed to investigate whether the correlations represent causality, thus whether the apoM/S1P complex directly affects BAT functionality. The cooling protocols used, fixed versus personalized, can potentially affect the BAT activity differently^40^ even though the participants in the two short-term cold studies are comparable regarding age, gender, BMI, and the associations were maintained after including the study design as a covariate in the linear regression analysis. Finally, insulin resistance is associated with a decrease in plasma apoM. In combination with our observations, it will be important to further explore whether such a reduction also can be linked to a decreased metabolic activity in BAT from humans with insulin resistance.

The effect of long-term cold exposure on plasma apoM and S1P was explored either by maintaining the core temperature at 36˚C or reducing it further to 33˚C in patients submitted after cardiac arrest. There were no effects of target temperature on apoM, S1P and lipid parameters, but only a general effect of cooling. Hence, cooling independent of the degree of cooling decreased apoM, S1P, TC, HDL-C, and LDL-C, whereas Triglycerides increased. The lack of difference between the cooling groups may be explained by the experimental setup. Hence, both groups were subjected to active cooling. The general decrease over time in apoM/S1P plasma levels after long-term cold may be supported by other studies as well. Thus, apoM and S1P plasma levels are decreased in sepsis and dengue fever both affecting vascular permeability^41–44^. The reduction could be explained by apoM being a negative acute-phase protein^45^ or simply as a consequence of a decrease in HDL-C^46^. In the present, LTC study patients were all hospitalized after cardiac arrest, a severe stress condition, and assisted by medical pressor treatment such as dobutamine, dopamine, and norepinephrine. Therefore, we cannot conclude whether the observed differences are an effect of the cold intervention, disease, or medical treatment. Additional studies in healthy individuals are therefore needed to elucidate whether the apoM/S1P complex is modulated due to long-term cold exposure.

The study has limitations. We see opposing effects of cold exposure on apoM and S1P plasma levels in the two short-term cold studies which may reflect the extent and method of cooling. It is known that various cooling protocols can induce different metabolic responses but no common guideline for investigations of BAT is implemented^40^. Both the applied fixed and personalized short-term cold protocol in the present study are standard methods. Our result underlines the need for such guidelines to translate the obtained data in such experiments into the relevant biological function of BAT in humans. Furthermore, quantification of activated BAT using a glucose tracer, i.e. ^18^F-FDG may be suboptimal, as cold exposure results in increased oxidation of fatty acids rather than glucose^14,34^. Further, cold-exposed mice increase BAT uptake of triglyceride-derived fatty acids^20^, mostly after selective lipolysis^47^. As described, apoM is linked to triglyceride metabolism and activity of BAT in mice^16^. Therefore, it cannot be excluded that using an FFA-tracer, preferentially incorporated within triglyceride to assess LPL-dependent lipolysis by BAT, could bring other results in the future. However, studies on lipid metabolism during non-shivering short-term cold exposure are sparse^13,48^. Thus, further studies in lipid metabolism during non-shivering cold using lipid tracers are needed to explore BAT metabolism. In the present cohorts, shivering was only controlled by verbal feedback and not by electromyography. We cannot exclude individuals who were not subclinical shivering, but a subanalysis did not show any correlation between plasma apoM and S1P with glucose uptake in muscles (data not shown). Changes observed in plasma apoM and S1P levels after cooling may also be affected by a 2-h extended fasting period. However, the literature does not suggest that neither total plasma apoM nor S1P concentrations are affected by fasting^24,49^. Our study did also not allow to investigate whether BAT-negative versus BAT-positive individuals have different plasma apoM levels. With a personalized cooling protocol, it is expected that 95% of the participants will have BAT, therefor it will be needed with significantly more participants than in the present study. Finally, the study cohorts are small in size but comparable to other published studies including quantification of BAT in humans.

In conclusion, we show that fixed versus personalized cooling procedures affect apoM and S1P plasma levels and, interestingly, are positively associated with parameters of BAT metabolic activity in humans. To our knowledge, the role of S1P signaling in human BAT is unknown. FTY720, an S1P analog, is an FDA-approved drug for multiple sclerosis^50^. Currently, drugs targeting the S1P receptor system are directed towards diseases involving the immune system^50,51^. It would be highly relevant to investigate whether drugs targeting the S1P signaling pathway in BAT could lead to increased vascular permeability and uptake of triglycerides augmenting energy expenditure and contributing to the prevention of adiposity and insulin resistance.

## Materials and methods

### Study setup and participants

Three different studies were included and conducted as described below (see also Fig. 1).

The STC-CPH cohort was conducted at Rigshospitalet and the University of Copenhagen. Healthy young white Caucasian men (age 18–33 years, body mass index (BMI) 20–27 kg/m^2^) were enrolled. Some of the participants were from a cohort published earlier^52^. Exclusion criteria were daily prescribed drugs and weekly alcohol and smoking consumption above 21 and 20 units, respectively. All 15 participants had given written informed consent. The study was approved by The National Committee on Health Research Ethics in Denmark (H-1–2013-064).

The STC-LEI cohort was conducted at the Alrijne Hospital in Leiderdorp, the Netherlands (Netherlands Trial Register 2473). Healthy Dutch South Asian and matched Dutch white Caucasian males with a lean phenotype (BMI < 25 kg/m^2^) matched for BMI and between the age of 18 and 28 years were recruited. Participants underwent medical examination including blood sampling and an oral glucose tolerance test for exclusion of type 2 diabetes. Besides type 2 diabetes, exclusion criteria were e.g. extensive exercise and smoking. Written informed consent from all participants was obtained and the study was approved by appropriate authorities^6^. The study has been described in detail by Bakker et al.^6^ and Hoeke et al.^48^. For the present study, we could include 10 white Caucasians and 10 South Asians.

The LTC cohort, included 44 participants from the single-center study at Rigshospitalet, Copenhagen University Hospital, Denmark (in total n = 171) as part of the Target Temperature Management (TTM) 33ºC versus 36ºC after out-of-hospital cardiac arrest (NCT01020916)^53^. The exclusion criteria for the TTM trial were in-hospital or presumes non-cardiac cause of arrest, severe comorbidity, admission temperature < 30 °C, and persistent cardiogenic shock ^21,54^. For the present study, additional exclusion criteria were women, age > 70 years, acute myocardial infarction, ischemic heart disease, arrhythmia, previous cardiac arrest, hypertension, diabetes, dialyzes, pulseless electrical activity plus asystole, and former percutaneous coronary intervention. These additional criteria were selected to align with the short-term cold studies (e.g. only men) as well as excluding diseases known to affect apoM such as diabetes and dialysis^55,56^. The TTM trial was designed as a multicenter, prospective investigator-initiated, randomized, parallel-group, and assessor-blinded trial including 36 intensive care units in Europe and Australia investigating the outcome of cooling patients to a core temperature of either 33 °C or 36 °C after cardiac arrest^21^. The LTC study was approved by the Ethics Committee of the Capital Region Copenhagen (H-1-2010-059) and the Danish Data Protection Agency, and written informed consent was in all cases obtained from the patients next of kin and general practitioner and by the patient if regaining consciousness after cardiac arrest^53,57^. Included patients were > 18 years of age and resuscitated after out of hospital cardiac arrest remaining unconscious (Glasgow Coma Score < 8) after sustained return to spontaneous circulation > 20 min.

All studies were conducted according to the principles of the Declaration of Helsinki.

### Procedure for cold exposure

In STC-CPH, participants fasted overnight. Two hours before the PET/CT scan the participants were put in a water-perfused cooling vest covering the upper body with a continuous flow of water at a fixed temperature of 14 °C (fixed cooling protocol^40^). One hour before a 2-bed PET/CT scan ^18^F-FDG was injected intravenously (97–100 MBq, mean ± SD: 99.07 ± 0.96 MBq). The 5-min per bed PET scans were performed on a Biograph TruePoint (Siemens, Knoxville, TN) with a low dose CT and 3D-OSEM reconstruction with PSF using 3 iterations 21 subsets and a 2 mm FWHM Gaussian filter (2 × 2x3 mm voxels). Heparin blood samples were collected just before and after 2 h of cold induction. Participants were monitored for shivering (observed by the researcher or/and self-reported by the participant) due to the cold exposure. BAT parameters were analyzed using Mirada RTx 1.0.2 (Mirada Medical Ltd, Oxford, UK) analysis to calculate standardized uptake value (SUV) maximum, mean, and BAT volume on PET.

In STC-LEI, participants underwent a 10 h overnight fast. To activate BAT the participants were imbedded between two water perfused cooling blankets. Once shivering was detected visually by researchers and self-reported by the participants, water temperature increased and a personalized cooling protocol^40^ started for 2 h. After 1 h 2 MBq/kg ^18^F-FDG was injected intravenously, and after one more hour of cold exposure, the PET/CT scan was performed for BAT quantification. Blood samples were collected at thermoneutral before cold and at time 110 min after cold induction^6^. BAT parameters were analyzed using the Beth Israel plugin for the FIJI program as described elsewhere^58–60^.

BAT was quantified using an SUV mean threshold at > 1.5 g/ml, Hounsfield units from -250 to -10 and anatomic position defined from the vertebrae C3 to T7 in participants from both STC-CPH and STC-LEI. We observed one individual with a low amount of BAT, but all individuals had visually detected BAT, and we did not observe any BAT negative individuals.

In LTC, patients were randomized to a core body temperature of either 33 or 36 °C for 24 h after cardiac arrest. The protocol included a 4 h induction period to achieve the target temperature. Hereafter, the target temperature was maintained for 24 h with subsequent rewarming to 37 °C with a maximum of 0.5 °C/hour. Plasma samples were taken before cold induction and 28 h after (4 h of induction period + 24 h of cold). Cooling was achieved using Thermowrap^21,53^.

#### Animal experiment

Wild-type female mice (C57B6) were randomized and housed at 23 ℃ (n = 8) or 4 ℃ (n = 8) for 16 h, at the Panum Institute (University of Copenhagen) in a temperature-controlled facility with a 12-h dark/light cycle and fed a standard chow diet. Blood samples were taken after 16 h of housing at 23 ℃ or 4 ℃ and blinded for plasma analysis. All procedures were approved by the Animal Experiments Inspectorate, Ministry of Justice, Denmark, and reported according to the ARRIVE guidelines.

### Ethic Declarations

All animal experiments and methods used in our animal experiments were performed in accordance with the relevant guidelines and regulations.

### Plasma analysis

Plasma S1P and apoM were measured as previously described^17,28^ and^23^, respectively.

In the STC-CPH and LTC studies plasma lipids TC, HDL-C, LDL-C, and Trig were measured at the Department of Clinical Biochemistry, Rigshospitalet, Denmark. The lipids were measured using standardized enzymatic absorption photometry assays on an automated analyzer (Cobas 8000, Roche) according to the manufacturer´s instructions. In the STC-LEI study lipids were measured according to Hoeke et al.^48^ and FFA according to Bakker et al.^6^. FFA was measured by an automated spectrophotometer (ABX Pentra 400 autoanalyzer) by enzymatic kit (WAKO NEFA C Kit; TriChem Aps) in STC-CPH.

### Statistics

Statistics were performed in SPSS Statistics 22 and 25. A paired t-test or a Wilcoxon t-test were performed between groups depending on whether data were normally distributed or not. Correlations were performed with Pearson or Spearman´s test depending on whether data were normally distributed. For analysis of the association between changes in plasma apoM or S1P, the STC-CPH and STC-LEI were combined after testing for homogeneity and interaction. In the linear regression analysis, the study protocol was included as a covariate. The effect of temperature and time in the LTC study was analyzed with a Two-Way Mixed ANOVA. A p-value < 0.05 was considered significant. Student´s t-test was used to compare groups in animal studies.

## Supplementary Information

Supplementary Information.

## Data Availability

The datasets generated during and/or analyzed during the current study are available from the corresponding author on reasonable request.
