# Supplementary material for: Association of apolipoprotein M and sphingosine-1-phosphate with brown adipose tissue after cold exposure in humans
Source: Sci Rep. 2022 Nov 5;12:18753. doi: 10.1038/s41598-022-21938-2 (PMC9637161; doi:10.1038/s41598-022-21938-2)
Supplement: Supplementary file 1 — Supplementary Information. [file 41598_2022_21938_MOESM1_ESM.docx]

**Supplementary material**

**Association of the apolipoprotein M and sphingosine-1-phosphate complex with brown adipose tissue after cold exposure in humans**

Anna Borup^1,6^, Ida Donkin^2^, Mariëtte R. Boon^3^, Martin Frydland^4^, Borja Martinez-Tellez^3^, Annika Loft^5^, Sune H. Keller^5^, Andreas Kjaer^5^, Jesper Kjaergaard^4^, Christian Hassager^4^, Romain Barrès^2^, Patrick C.N. Rensen^3^, Christina Christoffersen^1,6,7^

**Affiliations**

^1^Department of Clinical Biochemistry, Rigshospitalet, Copenhagen, Denmark, ^2^Novo Nordisk Foundation Center for Basic Metabolic Research, Faculty of Health and Medical Sciences, University of Copenhagen, Copenhagen, Denmark, ^3^Department of Medicine, Division of Endocrinology, and Einthoven Laboratory for Experimental Vascular Medicine, Leiden University Medical Center, Leiden, The Netherlands, ^4^Department of Cardiology, Rigshospitalet, Copenhagen, Denmark, ^5^Department of Clinical Physiology, Nuclear Medicine & PET and Cluster for Molecular Imaging, Rigshospitalet and University of Copenhagen, Copenhagen, Denmark,^6^Department of Biomedical Sciences, University of Copenhagen, Denmark, ^7^Department of Clinical Biochemistry, Bispebjerg Hospitalet, Denmark.

**Corresponding author**

Christina Christoffersen, MD, PhD, DMSc

Associated Professor

Department of Clinical Biochemistry

Rigshospitalet

Blegdamsvej 9

2100 Copenhagen

Denmark

Email: christina.christoffersen@regionh.dk

**
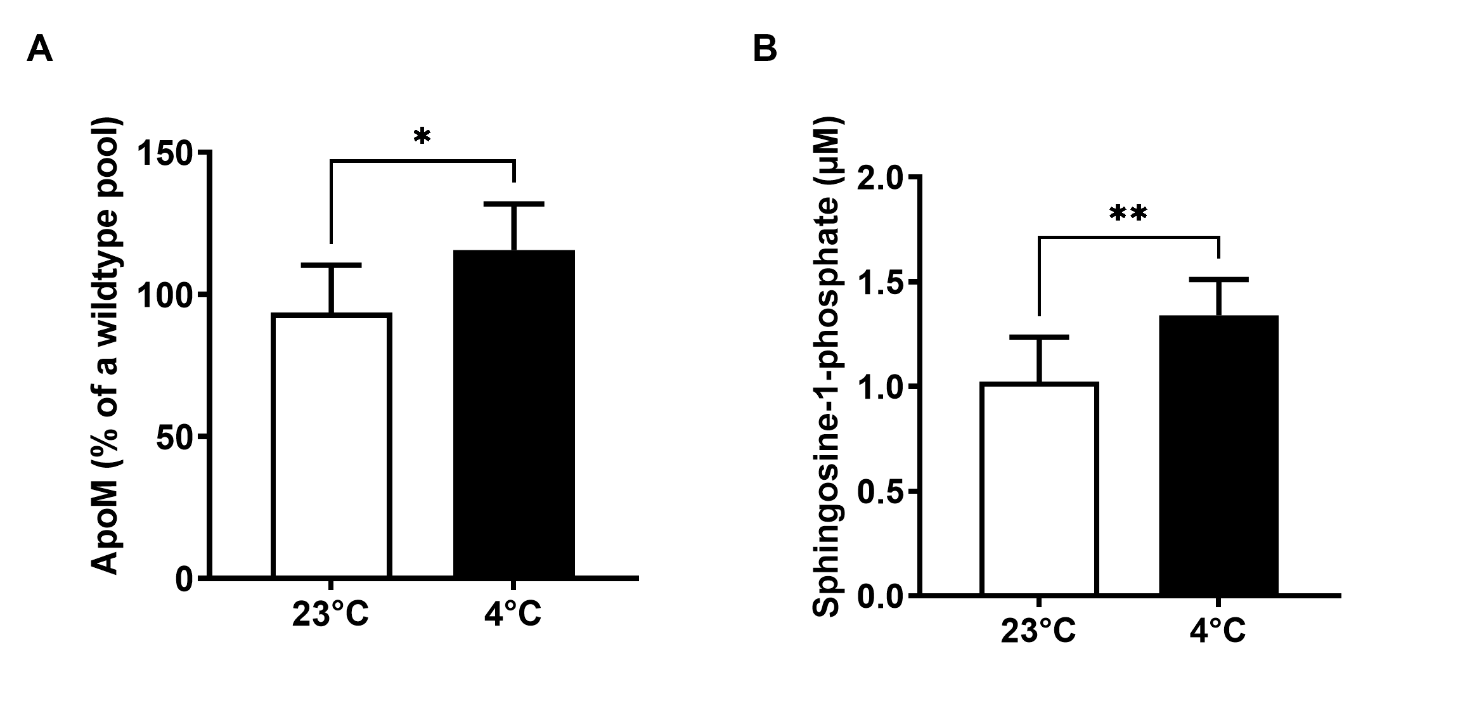
Supplementary figure S1.** Plasma apoM (A) and S1P (B) in wild-type mice exposed to 23°C (n=8) or 4°C (n=8) for 16 hours. Plasma apoM is reported as percent of a wild-type plasma pool used for calibration. Data is presented as mean (SD). *p<0.05; **p<0.01.

**
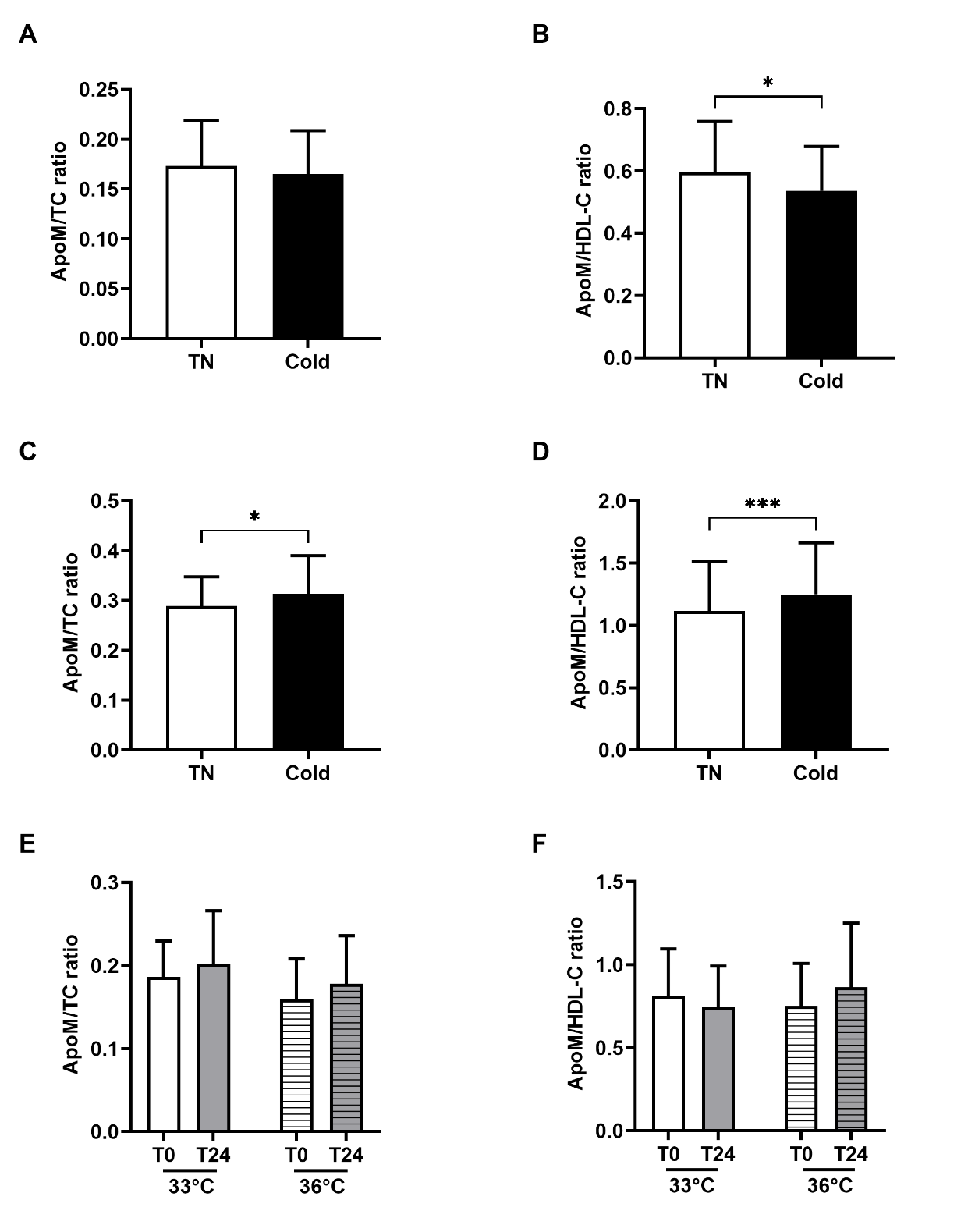
Supplementary figure S2.** ApoM/lipid ratios calculated for determining whether the change observed in apoM plasma levels could be explained by changes in lipid levels. ApoM/TC (A) and apoM/HDL-C (B) ratio in STC-CPH. ApoM/TC (C) and apoM/HDL-C (D)ratio in STC-LEI. ApoM/TC (E) and apoM/HDL-C (F) ratio in LTC. TC = total cholesterol, HDL-C = HDL cholesterol. Thermoneutral (TN) and cold refers to pre-cooling and after cold exposure (2 hours) in STC-CPH (n=15) and STC-LEI (n=19). In LTC samples were taken at 0 and after 2 hours at either 33°C (intensified cold, n=20) or 36°C (cold maintenance, n=24). Data presented as mean (SD). *p<0.05; ***p<0.001. STC-CPH, short-term cold, Copenhagen; STC-LEI, short-term cold, Leiderdorp; LTC, long-term cold.


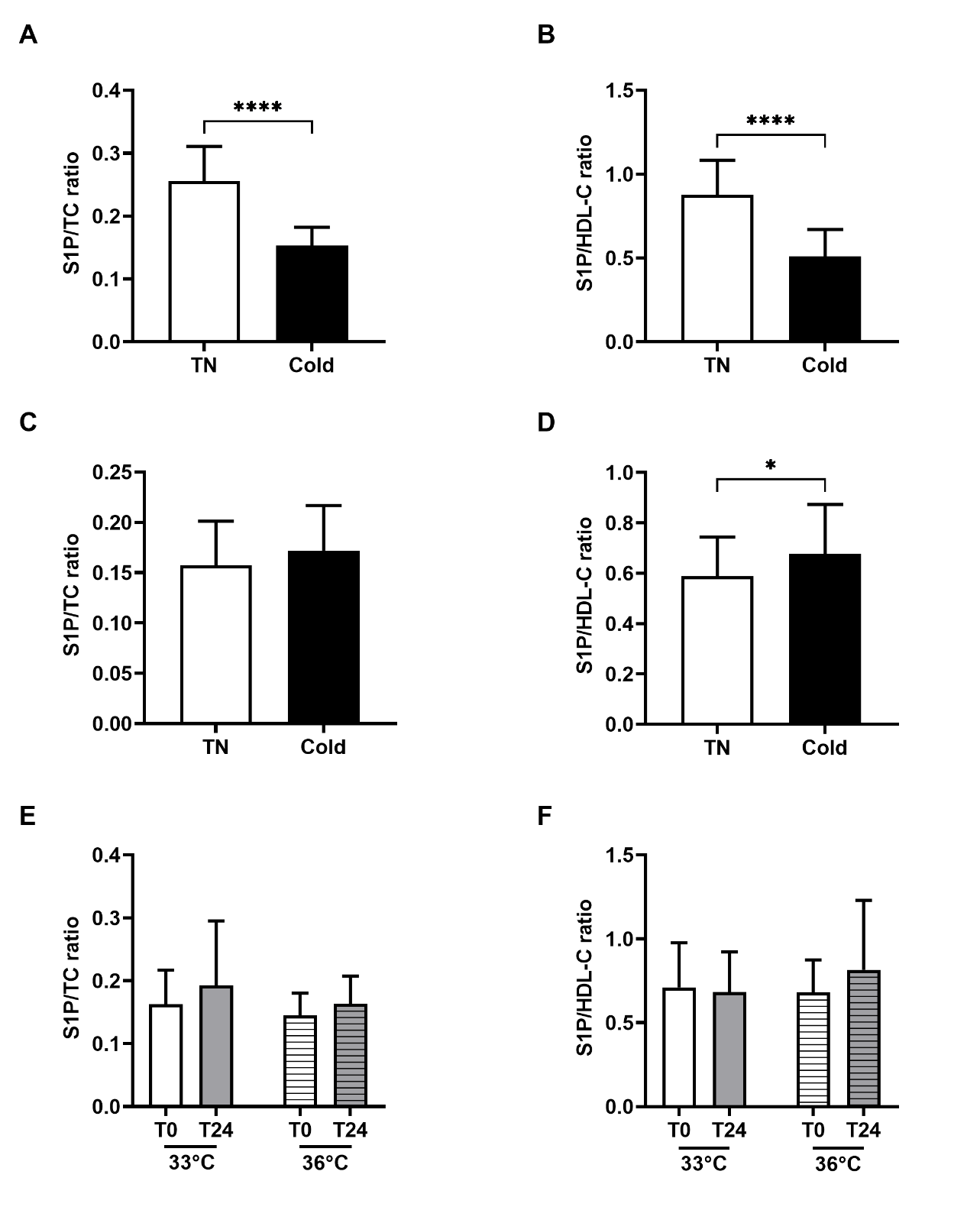


**Supplementary figure S3.** S1P/lipid ratios calculated for determining whether the change observed in S1P plasma levels could be explained by changes in lipid levels. S1P/TC (A) and S1P/HDL-C (B) ratio in STC-CPH. S1P /TC (C) and S1P /HDL-C (D) ratio in STC-LEI. S1P /TC (E) and S1P /HDL-C (F) ratios in LTC. TC = total cholesterol, HDL-C = HDL cholesterol. Thermoneutral (TN) and cold refers to pre-cooling and after cold exposure (2 hours) in STC-CPH (n=15) and STC-LEI (n=19). In LTC samples were taken at 0 and after 2 hours at either 33°C (intensified cold, n=20) or 36°C (cold maintenance, n=24). Data presented as mean (SD). *p<0.05; ****p<0.0001. STC-CPH, short-term cold, Copenhagen; STC-LEI, short-term cold, Leiderdorp; LTC, long-term cold.

**Supplementary Table S1**. **General characteristics on the three studies included.**

| **Study** | **Age**  **(years)** | **Weight**  **(kg)** | **BMI**  **(kg/m^2^)** |
| --- | --- | --- | --- |
| *STC-CPH* |  |  |  |
| All (n=15) | 24.3 (4.6) | 77.7 (7.8) | 23.2 (1.8) |
| *STC-LEI* |  |  |  |
| All (n=20) | 24.4 (2.8) | 70.6 (9.5) | 21.9 (1.8) |
| Cau (n=10) | 25.2 (2.7) | 75.6 (6.8) | 22.3 (1.4) |
| SA (n=10) | 23.7 (2.8) | 65.6 (9.4) | 21.4 (2.2) |
| *LTC* |  |  |  |
| 33°C (n=20) | 55.7 (10.3) | - | - |
| 36°C (n=24) | 53.3 (9.9) | - | - |

Data are presented as mean (SD). STC-CPH; short-term cold, Copenhagen. STC-LEI; short-term cold, Leiderdorp. LTC; long-term cold. Cau; White Caucasians. SA; south Asian**s.**

**Supplementary Table S2. Total cholesterol, HDL-cholesterol, LDL-cholesterol and triglycerides in plasma.**

| **Study** | **Total cholesterol**  **(mmol/L)** | | **HDL-cholesterol**  **(mmol/L)** | | **LDL-cholesterol**  **(mmol/L)** | | **Triglyceride**  **(mmol/L)** | |
| --- | --- | --- | --- | --- | --- | --- | --- | --- |
|  | **TN** | **Cold** | **TN** | **Cold** | **TN** | **Cold** | **TN** | **Cold** |
| *STC-CPH* |  |  |  |  |  |  |  |  |
| All (n=15) | 4.33 (0.70) | 4.13 (0.67) | 1.27 (0.22) | 1.28 (0.22) | 2.65 (0.71) | 2.55 (0.60) | 0.99 [0.63] | 0.84 [0.51] |
| *STC-LEI* |  |  |  |  |  |  |  |  |
| All (n=19) | 3.74 (0.89] | 4.01 (0.87)** | 0.99 (0.18) | 1.02 (0.20) | 2.31 (0.85) | 2.46 (0.80)* | 1.25 [0.62] | 1.48 [0.67]** |
|  |  |  |  |  |  |  |  |  |
|  | **T0** | **T24** | **T0** | **T24** | **T0** | **T24** | **T0** | **T24** |
| *LTC* |  |  |  |  |  |  |  |  |
| 33°C (n=20) | 4.87 (1.09) | 4.06 (1.28) | 1.16 (0.30) | 1.07 (0.24) | 3.08 (1.29) | 1.94 (1.04) | 1.08 [0.94] | 1.76 [2.01] |
| 36°C (n=24) | 5.14 (1.09) | 4.00 (0.88) | 1.13 (0.29) | 0.89 (0.33) | 3.29 (1.00) | 2.00 (0.74) | 1.11 [0.55] | 1.97 [1.52] |

Data are presented as mean (SD) if normally distributed, otherwise as median [IR]. In STC-CPH and STC-LEI cold refer to 2 hours cold exposure. In LTC samples were taken at 0 (T0) and after 24 (T24) hours at either 33˚C (intensified cold) or 36˚C (cold maintenance). Data were analyzed using paired t-test or Wilcoxon signed rank test for normally or not normally distributed data, respectively. In LTC data was analyzed with a two-way mixed ANOVA including temperature group and time as variables. For all parameters only an effect of time was found (p<0.0005 for all). Data from STC-LEI has partly been presented in Hoeke et al. ^21^. *p<0.01, ** p<0.005 TN vs Cold.

TN; thermoneutral. HDL; high density lipoprotein. LDL; low density lipoprotein. STC-CPH; short-term cold, Copenhagen. STC-LEI; short-term cold, Leiderdorp. LTC; long-term cold.

**Supplementary Table S3. Plasma free fatty acids.**

| **Study** | **TN** | **Cold** |
| --- | --- | --- |
| *STC-CPH* |  |  |
| All (n=15) | 0.54 (0.31) | 0.81 (0.41) |
| *STC-LEI* |  |  |
| All (n=18^†^) | 0.84 (0.36) | 1.02 (0.34)* |

Data are presented as mean (SD). Cold refers to 2 hours after start of cold exposure. Data are presented in mmol/L. Data were analyzed using paired t-test. Data from STC-LEI has partly been presented in ^6^. * p<0.005 TN vs Cold. ^†^Missing value for one participant. TN; thermoneutral. STC-CPH; short-term cold, Copenhagen. STC-LEI; short-term cold, Leiderdorp.

**Supplementary Table S4. Correlations between delta plasma apoM, delta plasma S1P and BAT after 2 hours cold exposure**

|  | **STC-CPH (n=11)** | | **STC-LEI (n=19)** | | |
| --- | --- | --- | --- | --- | --- |
|  | ΔapoM (µM) | ΔS1P (µM) | | ΔapoM (µM) | ΔS1P (µM) |
| BAT volume (cm^2^) | r=0.384, NS | r=0.316, NS | | r=0.376, NS | r=0.147, NS |
| BAT metabolic activity  (SUVmean*vol) | r=0.357, NS | r=0.295, NS | | r=0.440, NS | r=0.077, NS |
| SUVmean (g/ml) | r=0.094, NS | r=0.090, NS | | r=0.318, NS | r=-0.079, NS |
| SUVmax (g/ml) | r=-0.377, NS | r=-0.379, NS | | r=0.225, NS | r=-0.205, NS |

Correlations were performed with Pearson test. NS, non-significant. STC-CPH; short-term cold, Copenhagen. STC-LEI; short-term cold, Leiderdorp.
